# Altered plasma cytokine levels in acute and chronic central serous chorioretinopathy

**DOI:** 10.1111/aos.14547

**Published:** 2020-07-23

**Authors:** Izabella Karska‐Basta, Weronika Pociej‐Marciak, Michał Chrząszcz, Agnieszka Kubicka‐Trząska, Bożena Romanowska‐Dixon, Marek Sanak

**Affiliations:** ^1^ Faculty of Medicine Department of Ophthalmology Clinic of Ophthalmology and Ocular Oncology Jagiellonian University Medical College Kraków Poland; ^2^ Faculty of Medicine Department of Internal Medicine Molecular Biology and Clinical Genetics Unit Jagiellonian University Medical College Kraków Poland

**Keywords:** central serous chorioretinopathy, choroidal thickness, cytokines, hypertension, inflammation, interleukins, VEGF

## Abstract

**Purpose:**

To evaluate plasma levels of selected cytokines and investigate their correlation with choroidal thickness (CT) in patients with acute and chronic central serous chorioretinopathy (CSC).

**Methods:**

We enrolled 30 patients with acute CSC, 30 patients with chronic CSC and 20 controls. Plasma concentrations of 12 cytokines, interleukins IL‐8, IL‐1β, IL‐2, IL‐4, IL‐5, IL‐6, IL‐10 and IL‐12 p70, granulocyte‐macrophage colony‐stimulating factor, interferon‐γ, tumour necrosis factor‐α (TNF‐α) and vascular endothelial growth factor (VEGF), were measured using multiplex immunoassays. Differences in cytokine levels between groups were assessed. We also investigated correlations between cytokine levels and CT using swept‐source optical coherence tomography, as well as an association between plasma cytokine profile and systemic hypertension.

**Results:**

We noted differences in IL‐6 (p = 0.005), IL‐10 (p = 0.03), IL‐12 p70 (p = 0.028) and VEGF (p = 0.029) levels between groups. Pro‐inflammatory IL‐12 p70 and multidirectional IL‐10 cytokines were upregulated, while pro‐angiogenic VEGF was downregulated in chronic CSC as compared with controls (p = 0.005, p = 0.025 and p = 0.027, respectively). Interleukin‐6 (IL‐6) was upregulated in acute and chronic CSC (p = 0.030 and p = 0.005, respectively). Interleukin‐5 (IL‐5), IL‐6 and IL‐12 levels correlated with mean CT in acute CSC (p = 0.008, p = 0.003 and p = 0.044, respectively), while IL‐8, IL‐6 and TNF‐α plasma levels correlated with hypertension in chronic CSC (p = 0.005, p = 0.033 and p = 0.001, respectively).

**Conclusion:**

We provided new evidence for the possible role of plasma cytokines in the pathogenesis of CSC. Our results suggest that IL‐6 may be important in the pathophysiology of acute and chronic CSC. The association between inflammatory response and hypertension in patients with CSC was also confirmed.

## Introduction

Central serous chorioretinopathy (CSC) is the fourth most common retinopathy after age‐related macular degeneration (AMD), diabetic retinopathy and retinal vein occlusion (Wang et al. 2008). Progression of CSC over time may lead to vision disorder and deteriorated quality of life (Karska‐Basta et al. 2020). There are two types of CSC: acute and chronic, with a threshold of between 4 and 6 months adopted in most published reports to distinguish between both entities (Daruich et al. 2015). The disease is characterized by the presence of serous subretinal fluid, retinal pigment epithelial damage, as well as dilated choroidal vessels (so‐called pachyvessels) and their hyperpermeability on indocyanine green angiography (Jirarattanasopa et al. 2012; Cheung et al. 2019; van Haalen et al. 2020).

As the pathogenesis of CSC has not been fully elucidated (Daruich et al. 2015), there is currently no effective treatment (Pociej‐Marciak et al. 2016; Lee et al. 2019). Numerous studies have emphasized the key role of choroidal thickness (CT) in the pathogenesis of CSC, which is nowadays considered as one of the pachychoroid diseases (Kim et al. 2011; Sakurada et al. 2018).

Central serous chorioretinopathy (CSC) was reported to be a potential risk factor for coronary artery disease in men as well as an independent risk factor for ischaemic stroke, which could indicate an association with other cardiovascular diseases (Tsai et al. 2013; Chen et al. 2014). Moreover, persistently elevated levels of plasma cytokines have been reported in patients with a history of vascular dysfunction and cardiovascular diseases (Sprague & Khalil 2009). However, data on the links between the upregulation of plasma cytokine levels and CSC are inconsistent (Lim et al. 2010; Terao et al. 2018). There have been only few studies indicating that dysregulation of aqueous humour (AH) cytokine levels may be associated with CSC (Shin & Lim 2011; Terao et al. 2018). To date, only one report describing the lack of an association between plasma cytokine levels [vascular endothelial growth factor (VEGF) and interleukin IL‐8] and CSC has been published (Lim et al. 2010).

The aim of this study was to analyse the potential role of plasma cytokines: interleukins IL‐8, IL‐1β, IL‐2, IL‐4, IL‐5, IL‐6, IL‐10 and IL‐12 p70, granulocyte‐macrophage colony‐stimulating factor (GM‐CSF), interferon‐γ, tumour necrosis factor‐α (TNF‐α) and VEGF in the pathogenesis of acute and chronic CSC.

## Materials and Methods

### Study population

We screened 87 white patients over the age of 18 years with a diagnosis of CSC, of whom 49 men and 11 women were finally included in this case–control study. The research was conducted at the Clinic of Ophthalmology and Ocular Oncology in Kraków, Poland, from November 2017 to the end of May 2018. The diagnosis of CSC was based on characteristic fundus findings, fluorescein angiography (FA), fundus autofluorescence and swept‐source optical coherence tomography (SS‐OCT). General exclusion criteria were as follows: any acute illness, C‐reactive protein levels higher than 10 mg/l, renal or hepatic dysfunction, cancer, acute myocardial infarction or stroke, anticoagulation and corticosteroid treatment. Ocular exclusion criteria included uveitis, choroidal neovascularization (CNV), retinal vasculitis, polypoidal choroidal vasculopathy or other maculopathies causing macular exudation. We excluded 27 patients due to comorbidities. The control group comprised 20 individuals from the general population sample, who were matched for age, sex, smoking and hypertension. This study was conducted in accordance with the tenets of the Declaration of Helsinki. The study was approved by Jagiellonian University Bioethical Committee (no. 122.6120.266.2016), and all patients provided written informed consent to participate in the study.

### Clinical examination

All patients and controls underwent a complete ophthalmological examination, including best‐corrected visual acuity (BCVA) assessment, fundus biomicroscopy and SS‐OCT (DRI OCT Atlantis, Topcon, Japan). Fluorescein angiography (FA) was performed only in the CSC group (SPECTRALIS, Heidelberg Engineering, Germany).

Acute CSC was diagnosed based on the presence of typical clinical features and symptoms lasting less than 6 months. In the acute form, FA detected one or several leakage points at the level of the retinal pigment epithelium, while SS‐OCT revealed pigmented epithelial detachment or subretinal fluid (or both) as well as increased CT. When persistent subretinal fluid on SS‐OCT was observed for longer than 6 months and widespread areas of fluorescein leakage from the sites of retinal pigment epithelial damage were seen on FA, chronic CSC was diagnosed.

**Fig. 1:**
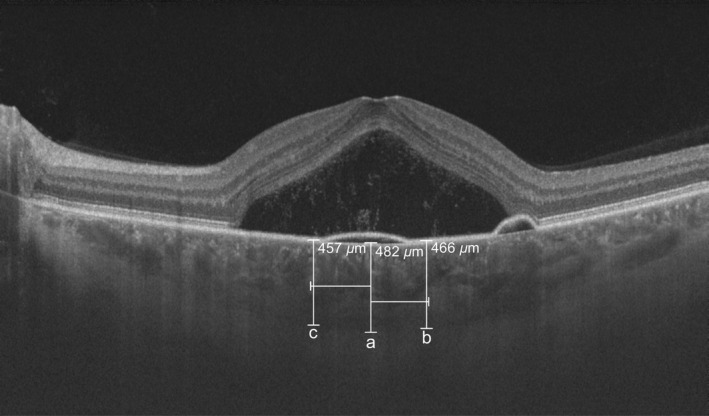
Horizontal swept‐source optical coherence tomography B‐scan of acute central serous chorioretinopathy. The assessment of choroidal thickness at three points: beneath the fovea (a), 750 *µ*m temporally (b) and 750 *µ*m nasally (c) from the fovea.

The assessment of CT was based on the method previously described by Branchini et al. (2013). We performed a horizontal SS‐OCT B‐scan, and CT was measured from the Bruch's membrane to the inner wall of the sclera at three points: beneath the fovea and then 750 *µ*m temporally and 750 *µ*m nasally from the fovea (Fig. 1). The average of these three measurements was considered as mean CT.

### Sample collection

Blood samples in all participants were obtained from the antecubital vein in the morning into Vacutainer (BD Life Sciences, Franklin Lakes, NJ, USA). The Magnetic Luminex Performance Assay kit for human high‐sensitivity cytokines (FCSTM09‐12; R&D Systems, Bio‐Techne, Minneapolis, MN, USA) was used to measure the concentrations of 12 different cytokines in blood plasma. Samples obtained from all participants were tested. The multiplex immunoassay contains premixed fluorogenic beads with monoclonal antibodies against GM‐CSF, interferon‐γ, IL‐8, IL‐1β, IL‐2, IL‐4, IL‐5, IL‐6, IL‐10, IL‐12 p70, TNF‐α and VEGF. The measurements were done according to the manufacturer's protocol using 1:2 diluted plasma and the xMAP analyser (Luminex Corporation, Austin, TX, USA). Bead‐trapped cytokines were detected by biotin–streptavidin sandwich immunocomplex fluorescence. The results were calculated using 7‐point standard curves and proprietary software, Milliplex Analyst version 5.1 (Merck, Darmstadt, Germany).

### Statistical analysis

Qualitative data were presented as counts and percentages. Quantitative data were shown as means and standard deviations for normally distributed variables and as medians and interquartile ranges (IQRs) otherwise. The normality of quantitative variables was tested using the Kolmogorov–Smirnov test. Intergroup comparisons of qualitative variables were made using the chi‐square test. The exact chi‐square test was used when expected frequencies in at least 20% of the cells were lower than 5. The Kruskal–Wallis test was used for intergroup comparisons of quantitative variables. When the comparison of the three groups resulted in a significant p‐value, it was followed by a pairwise comparison with Bonferroni's correction. The strength of a relationship between quantitative variables was assessed using the Spearman rho correlation coefficient.

A p‐value of <0.05 was considered significant. This study was powered to have an 80% probability of detecting a 15% difference in the mean CT level using a p‐value of 0.05. IBM SPSS Statistics 24 Version 25.0. (IBM Corp., Armonk, NY) for Windows was used for statistical analysis.

## Results

Our study group included 30 patients with acute CSC [25 men (83.3%)], 30 patients with chronic CSC [24 men (80%)] and 20 controls [11 men (55%)]. Treatment for CSC was applied in 10 patients (33.3%) with acute form and in all 30 patients (100%) with chronic form, depending on indications. Treatment modalities included half‐dose photodynamic therapy, subthreshold micropulse laser, oral mineralocorticoid receptor antagonists and topical nonsteroidal anti‐inflammatory drugs. No differences in sex distribution, age, smoking status or prevalence of hypertension were noted between patients with CSC and controls. The demographic and clinical characteristics as well as data on comorbidities in the study groups are presented in Table 1. There were no patients with diabetes, ulcerative colitis or obesity. We excluded 27 patients with comorbidities that might have affected the results (e.g. acute inflammatory disorders or any conditions associated with elevated C‐reactive protein levels). Any comorbidities that did not constitute an exclusion criterion are presented in Table 1. We did not observe any association between those underlying conditions and the obtained results.

**Table 1:** Demographic and clinical characteristics of patients with acute and chronic central serous chorioretinopathy and controls.

Parameter	Acute CSC (*n* = 30)	Chronic CSC (*n* = 30)	Control (*n* = 20)	p‐value
Female sex, *n* (%)	5 (17)	6 (20)	9 (45)	0.056
Age, years	42.7 ± 9.9	44.5 ± 6.1	39.2 ± 7.4	0.078
Hypertension, *n* (%)	11 (37)	6 (20)	4 (20)	0.260
Hashimoto's thyroiditis, *n* (%)	1 (3)	2 (6)	0 (0)	0.781
*Helicobacter pylori* infection, *n* (%)	0 (0)	6 (20)	0 (0)	0.007
Gout, *n* (%)	1 (3)	2 (6)	0 (0)	0.781
Ischaemic heart disease, *n* (%)	1 (3)	1 (3)	0 (0)	1.000
Smoking, *n* (%) (current, former)	7 (23)	8 (27)	7 (35)	0.658
CT, *µ*m	421.5 ± 85.3	406.1 ± 88.1	317.4 ± 61.4	<0.001
Affected eye, *n* (%)				
Right	14 (47)	10 (33)	–	0.225
Left	13 (43)	12 (40)	–	
Both eyes	3 (10)	8 (27)	–	
BCVA, *n* (%)				
0.5<*≤1.0	20 (67)	23 (77)	20 (100)	0.017
0.1≤*≤0.5	10 (33)	7 (23)	0	

Normally distributed variables are shown as mean ± SD.

BCVA = best‐corrected visual acuity, CSC = central serous chorioretinopathy, CT = choroidal thickness.

**Fig. 2:**
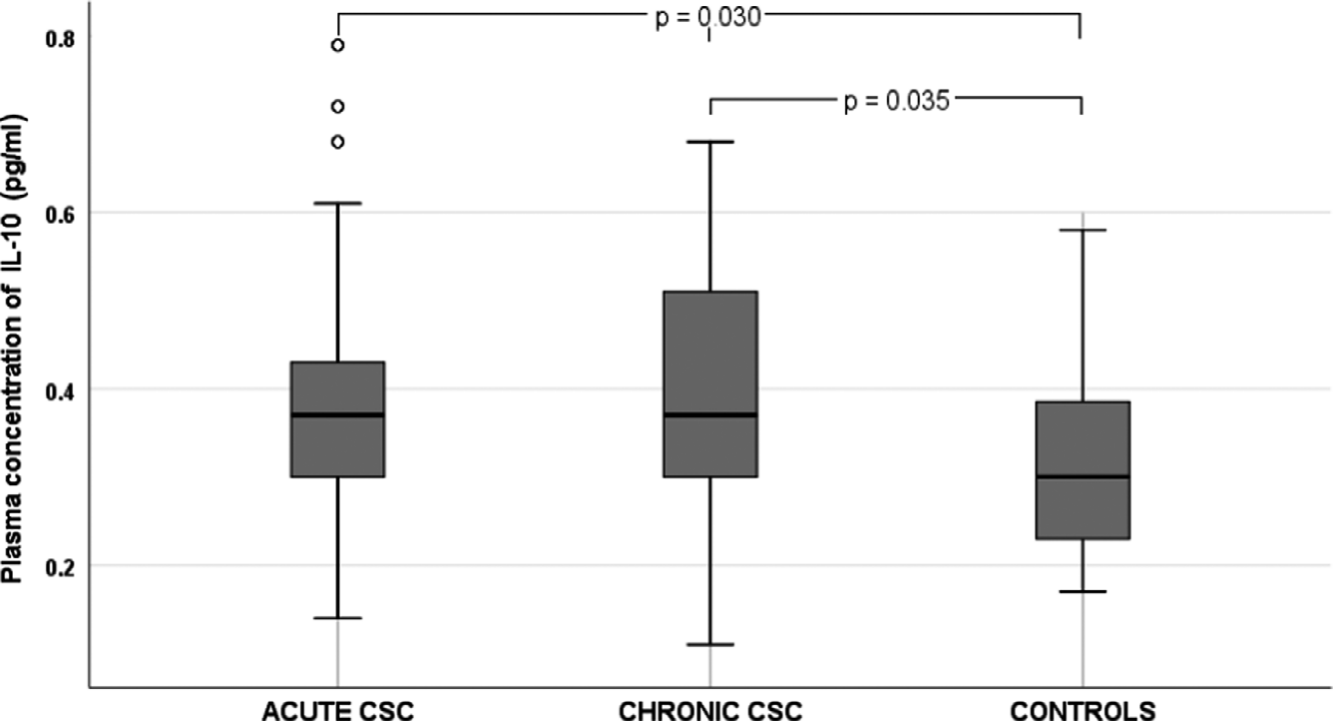
Box‐and‐whisker plot of plasma interleukin‐10 levels in patients with acute and chronic central serous chorioretinopathy and controls.

**Fig. 3:**
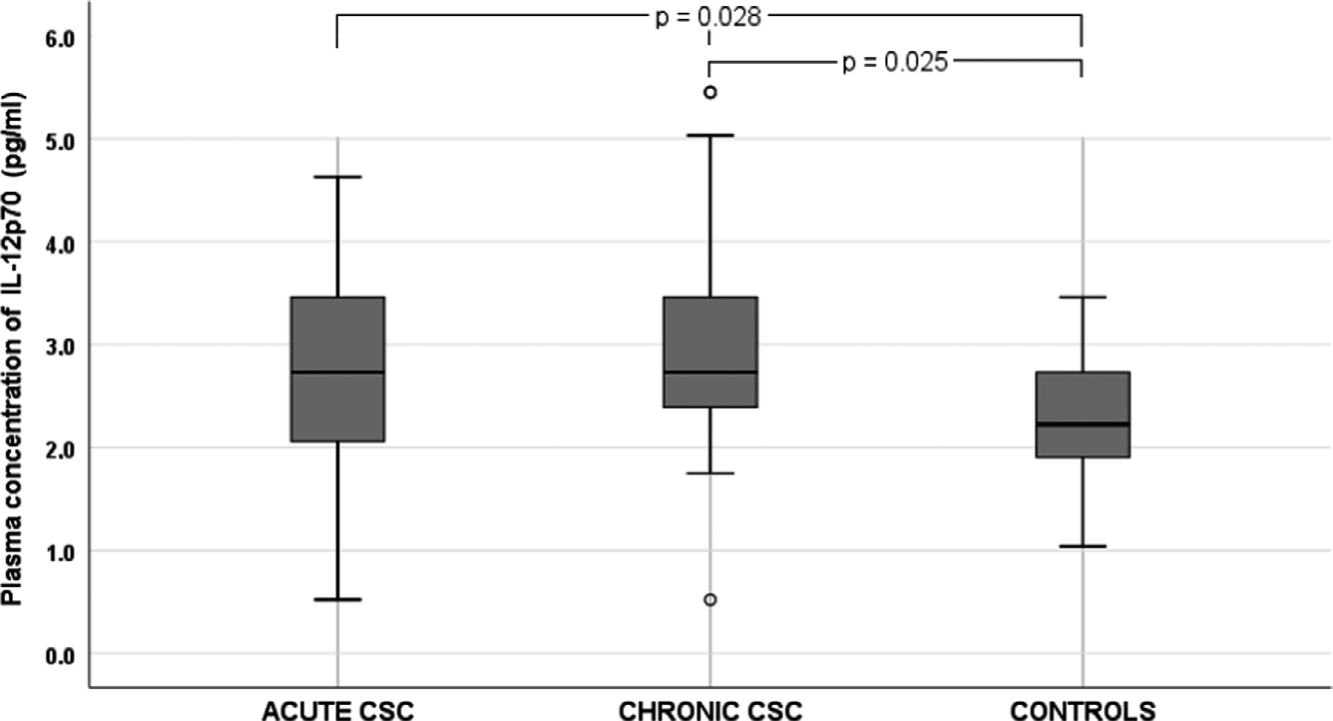
Box‐and‐whisker plot of plasma interleukin‐12 p70 levels in patients with acute and chronic central serous chorioretinopathy and controls.

**Fig. 4:**
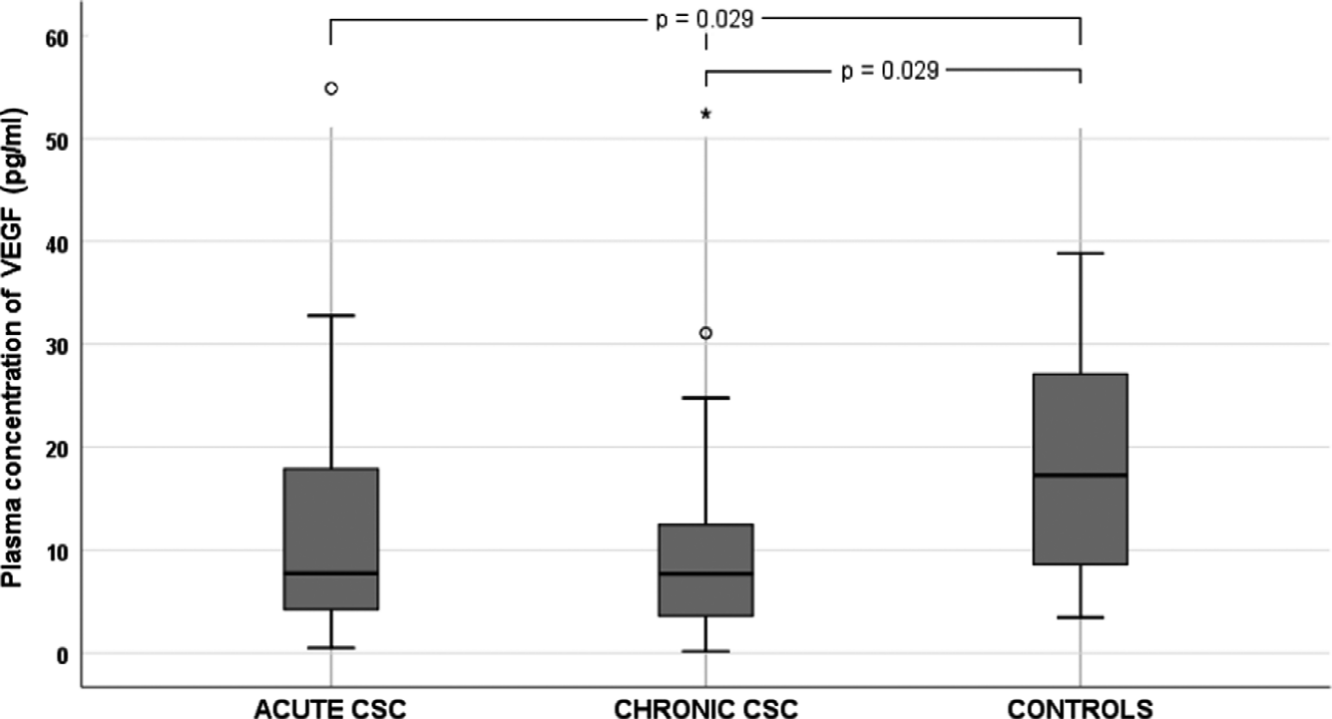
Box‐and‐whisker plot of plasma vascular endothelial growth factor levels in patients with acute and chronic central serous chorioretinopathy and controls.

**Fig. 5:**
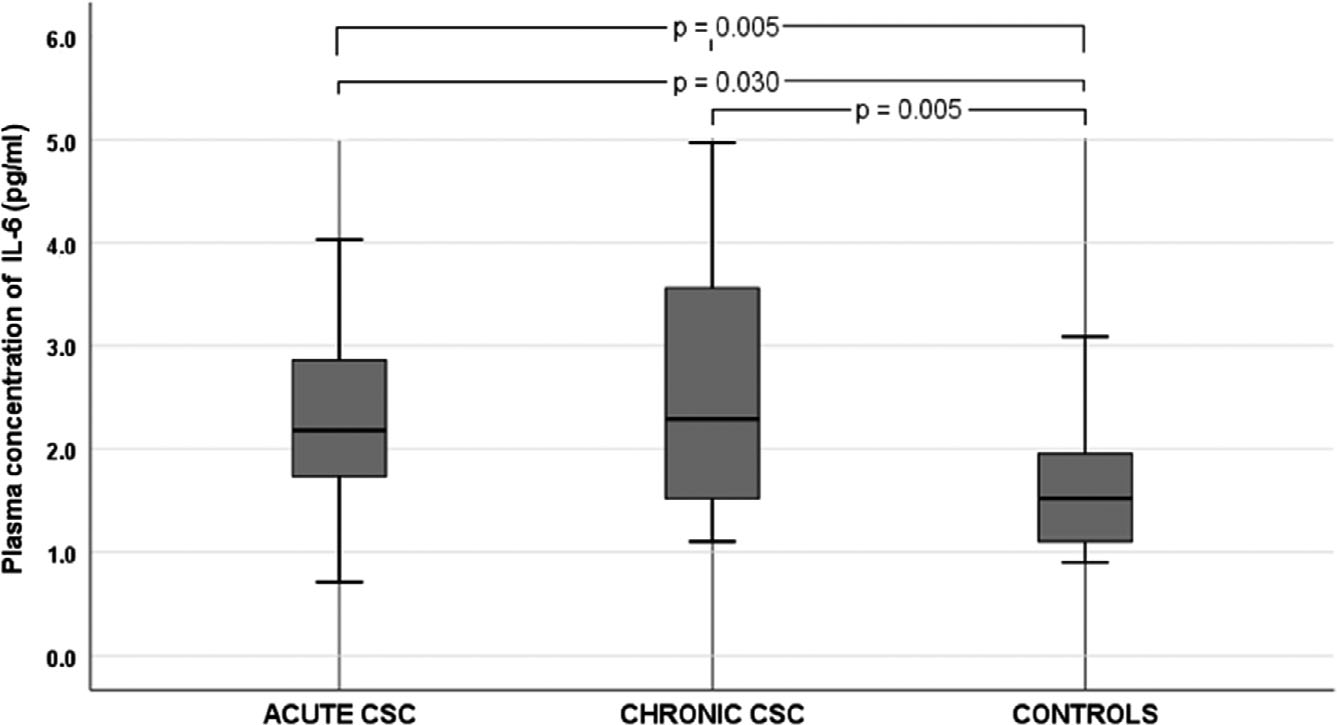
Box‐and‐whisker plot of plasma interleukin‐6 levels in patients with acute and chronic central serous chorioretinopathy and controls.

We noted significant differences in BCVA and mean CT between the three groups (Table 1). Among the 12 cytokines analysed, there were significant differences in plasma levels of IL‐6 (p = 0.005), IL‐10 (p = 0.030), IL‐12 p70 (p = 0.028) and VEGF (p = 0.029) between groups. The plasma levels of cytokines in CSC groups and controls are shown in Table 2. Plasma IL‐10 levels were significantly higher in patients with chronic CSC than in the control group (Fig. 2). Similarly, IL‐12 p70 levels were significantly higher in chronic CSC than in controls (Fig. 3). Interestingly, VEGF levels were significantly lower in patients with CSC than in controls (Fig. 4). Finally, patients with chronic and acute CSC had significantly higher plasma IL‐6 levels than the control group (Fig. 5). There were no significant differences in plasma cytokine levels between patients with acute and chronic CSC (data not shown).

**Table 2:** Plasma cytokine levels in patients with acute and chronic central serous chorioretinopathy and controls.

Cytokine	Acute CSC (*n* = 30)	Chronic CSC (*n* = 30)	Control (*n* = 20)	p‐value
TNF‐α, pg/ml	3.11 (2.57–3.79)	3.48 (3.03–3.94)	3.26 (2.65–3.79)	0.429
VEGF, pg/ml	**7.74 (4.23–17.87)**	**7.70 (3.58–12.46)**	**17.26 (8.59–27.10)**	**0.029**
IL‐1β, pg/ml	0.27 (0.20–0.36)	0.28 (0.18–0.41)	0.23 (0.19–0.35)	0.955
IL‐2, pg/ml	0.93 (0.55–1.33)	0.85 (0.70–1.50)	0.70 (0.55–0.93)	0.150
IL‐4, pg/ml	3.69 (0.51–5.31)	4.23 (2.06–7.43)	2.06 (1.01–5.31)	0.319
IL‐5, pg/ml	0.25 (0.20–0.30)	0.25 (0.20–0.35)	0.19 (0.15–0.27)	0.064
IL‐6, pg/ml	**2.18 (1.73–2.86)**	**2.29 (1.52–3.56)**	**1.52 (1.10–1.95)**	**0.005**
IL‐8, pg/ml	1.68 (1.29–2.12)	1.67 (1.36–2.68)	2.49 (1.68–3.28)	0.103
IL‐10, pg/ml	**0.37 (0.30–0.43)**	**0.37 (0.30–0.51)**	**0.30 (0.23–0.39)**	**0.030**
IL‐12 p70, pg/ml	**2.73 (2.06–3.46)**	**2.73 (2.39–3.46)**	**2.23 (1.91–2.73)**	**0.028**
GM‐CSF, pg/ml	0.25 (0.19–0.32)	0.30 (0.23–0.36)	0.32 (0.25–0.49)	0.114
Interferon‐γ, pg/ml	2.62 (1.52–3.84)	2.06 (1.52–3.43)	1.87 (1.52–2.24)	0.238

Data are shown as median (interquartile range). Significant correlations are presented in bold.

CSC = central serous chorioretinopathy, GM‐CSF = granulocyte‐macrophage colony‐stimulating factor, IL = interleukin, TNF‐α = tumour necrosis factor‐α, VEGF = vascular endothelial growth factor.

Correlations between mean CT and cytokine plasma levels in patients with acute CSC, chronic CSC and controls are summarized in Table 3. Of note, positive correlations were observed only in patients with acute CSC: for mean CT and plasma IL‐5, IL‐6 and IL‐12 p70 levels. In patients with acute and chronic CSC, we noted negative but nonsignificant correlations between mean CT and plasma VEGF levels. This was in contrast to the control group, which showed a positive correlation between these variables.

**Table 3:** Correlations between mean choroidal thickness and plasma cytokine levels in patients with acute and chronic central serous chorioretinopathy and controls.

Cytokine	Mean CT		
	Acute CSC (*n* = 30)	Chronic CSC (*n* = 30)	Control (*n* = 20)
TNF‐α	*r* = 0.004 p = 0.982	*r* = −0.245 p = 0.192	*r* = −0.075 p = 0.752
VEGF	*r* = −0.035 p = 0.857	*r* = −0.299 p = 0.108	*r* = 0.173 p = 0.466
IL‐1β	*r* = 0.093 p = 0.630	*r* = −0.136 p = 0.472	*r* = −0.155 p = 0.515
IL‐2	*r* = 0.453 p = 0.114	*r* = −0.154 p = 0.415	*r* = −0.116 p = 0.627
IL‐4	*r* = 0.283 p = 0.137	*r* = −0.079 p = 0.677	*r* = −0.024 p = 0.920
IL‐5	***r* = 0.485** **p = 0.008**	*r* = −0.228 p = 0.227	*r* = −0.087 p = 0.716
IL‐6	***r* = 0.532** **p = 0.003**	*r* = −0.295 p = 0.114	*r* = −0.110 p = 0.644
IL‐8	*r* = 0.235 p = 0.220	*r* = −0.261 p = 0.164	*r* = 0.229 p = 0.331
IL‐10	*r* = 0.297 p = 0.118	*r* = −0.150 p = 0.429	*r* = −0.018 p = 0.939
IL‐12 p70	***r* = 0.376** **p = 0.044**	*r* = 0.122 p = 0.522	*r* = 0.188 p = 0.428
GM‐CSF	*r* = 0.218 p = 0.256	*r* = −0.154 p = 0.417	*r* = 0.119 p = 0.618
Interferon‐γ	*r* = 0.250 p = 0.191	*r* = −0.141 p = 0.456	*r* = −0.188 p = 0.427

Data are presented as Spearman rho correlation coefficient; p < 0.05 was considered significant (bold).

CSC = central serous chorioretinopathy, GM‐CSF = granulocyte‐macrophage colony‐stimulating factor, IL = interleukin, TNF‐α = tumour necrosis factor‐α; VEGF = vascular endothelial growth factor.

Interestingly, only in the chronic CSC group, individuals with systemic hypertension had higher median (IQR) plasma levels of IL‐8 [2.95 pg/ml (2.53–3.47) versus 1.56 pg/ml (1.34–2.06); p = 0.005], IL‐6 [3.6 pg/ml (3.1–4.5) versus 2.1 pg/ml (1.4–3.0); p = 0.033] and TNF‐α [4.09 pg/ml (4.09–4.25) versus 3.33 pg/ml (2.95–3.79); p = 0.001], as compared with those without hypertension. There were no differences in the median (IQR) plasma levels of cytokines between patients with and without hypertension in the acute CSC group [IL‐8: 1.69 pg/ml (1.29–3.09) and 1.67 pg/ml (1.19–1.99), p = 0.830, respectively; IL‐6: 2.2 pg/ml (2.00–3.1) and 2.2 pg/ml (1.5–2.6), p = 0.635, respectively; TNF‐α: 3.18 pg/ml (2.57–4.09) and 3.03 pg/ml (2.57–3.79), p = 0.846, respectively]. Similarly, there were no differences in the median (IQR) levels of cytokines between patients with and without hypertension in the control group [IL‐8: 2.00 pg/ml (1.46–2.65) and 2.60 pg/ml (1.68–3.74), p = 0.385, respectively; IL‐6: 1.9 pg/ml (1.5–2.6) and 1.4 pg/ml (1.1–1.7), p = 0.064, respectively; TNF‐α: 3.03 pg/ml (2.29–3.33) and 3.33 pg/ml (2.73–3.94), p = 0.249, respectively]. To eliminate bias, an additional analysis to compare IL‐6, IL‐8 and TNF‐α levels in patients with hypertension between the groups with acute CSC, chronic CSC and controls was performed. The cytokine levels were highest in patients with chronic CSC. There were significant differences in the levels of IL‐6 and TNF‐α between groups (p = 0.042 and p = 0.005, respectively), while differences in IL‐8 had borderline significance (p = 0.053). The results are presented in Table 4.

**Table 4:** Plasma cytokine levels in participants with systemic hypertension according to the study group.

Cytokine	Acute CSC (*n* = 11)	Chronic CSC (*n* = 6)	Controls (*n* = 4)	p‐value
IL‐6	2.2 (2.0–3.1)	3.6 (3.1–4.5)	1.9 (1.5–2.6)	0.042*
				1.000^†^
				0.144^‡^
				0.111^§^
IL‐8	1.69 (1.29–3.09)	2.95 (2.53–3.47)	2.0 (1.46–2.65)	0.053*
TNF‐α	3.18 (2.57–4.09)	4.09 (4.09–4.25)	3.03 (2.29–3.33)	0.005*
				1.000^†^
				0.015^‡^
				0.018^§^

Data are shown as median (interquartile range). A p‐value of <0.05 was considered significant.

The p‐value for pairwise comparison was adjusted using the Bonferroni correction.

CSC = central serous chorioretinopathy, IL = interleukin, TNF‐α =_ _tumour necrosis factor‐α.

* Acute CSC versus chronic CSC versus controls.

^†^ Acute CSC versus controls.

^‡^ Chronic CSC versus controls.

^§^ Dcute CSC versus chronic CSC.

Most positive correlations between ILs were similar in patients with acute and chronic CSC. Interestingly, the control group showed the lowest number of significant correlations between the cytokines, and these correlations were also weaker than those in patients with CSC. The correlations between the plasma levels of all analysed cytokines in patients with acute and chronic CSC and controls are shown in Table 5.

**Table 5:** Correlations between plasma cytokine levels in patients with acute and chronic central serous chorioretinopathy and in controls.

Group\Parameter		IL‐1β	IL‐2	IL‐4	IL‐5	IL‐6	IL‐8	IL‐10	IL‐12 p70	TNF‐α	VEGF	GM‐CSF	Interferon‐γ
Acute CSC	IL‐1β	1.000											
	IL‐2	0.359	1.000										
	IL‐4	0.383^*^	0.852^**^	1.000									
	IL‐5	0.315	0.858^**^	0.794^**^	1.000								
	IL‐6	0.336	0.789^**^	0.750^**^	0.715^**^	1.000							
	IL‐8	0.369^*^	0.311	0.238	0.216	0.530^**^	1.000						
	IL‐10	0.406^*^	0.815^**^	0.913^**^	0.747^**^	0.682^**^	0.179	1.000					
	IL‐12 p70	0.365^*^	0.657^**^	0.675^**^	0.750^**^	0.445^*^	0.074	0.591^**^	1.000				
	TNF‐α	0.464^**^	0.276	0.167	0.256	0.252	0.294	0.195	0.180	1.000			
	VEGF	0.507^**^	0.042	0.135	−0.041	0.207	0.309	0.123	0.116	0.407^*^	1.000		
	GM‐CSF	0.478^**^	0.501^**^	0.414^*^	0.494^**^	0.364^*^	0.150	0.317	0.549^**^	0.701^**^	0.476^**^	1.000	
	Interferon‐γ	0.429^*^	0.625^**^	0.619^**^	0.498^**^	0.663^**^	0.521^**^	0.542^**^	0.436^*^	0.404^*^	0.425^*^	0.555^**^	1.000
Chronic CSC	IL‐1β	1.000											
	IL‐2	0.245	1.000										
	IL‐4	0.389^*^	0.879^**^	1.000									
	IL‐5	0.224	0.919^**^	0.821^**^	1.000								
	IL‐6	0.328	0.901^**^	0.811^**^	0.857^**^	1.000							
	IL‐8	0.341	0.170	0.210	0.071	0.224	1.000						
	IL‐10	0.365^*^	0.825^**^	0.824^**^	0.754^**^	0.821^**^	0.273	1.000					
	IL‐12 p70	0.305	0.630^**^	0.738^**^	0.543^**^	0.582^**^	0.216	0.698^**^	1.000				
	TNF‐α	0.166	0.511^**^	0.498^**^	0.400^*^	0.588^**^	0.454^*^	0.465^**^	0.500^**^	1.000			
	VEGF	0.242	−0.148	−0.121	−0.156	−0.129	0.351	−0.005	−0.065	0.205	1.000		
	GM‐CSF	0.104	0.103	0.222	0.055	−0.111	0.089	0.233	0.296	0.038	0.310	1.000	
	Interferon‐γ	0.265	0.848^**^	0.734^**^	0.839^**^	0.871^**^	0.119	0.839^**^	0.552^**^	0.363^*^	−0.096	−0.062	1.000
Controls	IL‐1β	1.000											
	IL‐2	0.138	1.000										
	IL‐4	0.217	0.578^**^	1.000									
	IL‐5	0.303	0.798^**^	0.546^*^	1.000								
	IL‐6	−0.016	0.858^**^	0.688^**^	0.790^**^	1.000							
	IL‐8	−0.057	−0.239	−0.136	−0.075	−0.201	1.000						
	IL‐10	0.183	0.738^**^	0.201	0.576^**^	0.469^*^	−0.284	1.000					
	IL‐12 p70	0.115	0.279	0.204	0.333	0.164	0.202	0.268	1.000				
	TNF‐α	0.180	0.139	0.298	0.035	0.026	0.363	0.013	0.266	1.000			
	VEGF	0.075	−0.193	−0.149	−0.164	−0.242	0.524^*^	−0.152	−0.142	0.206	1.000		
	GM‐CSF	−0.037	0.601^**^	0.111	0.390	0.399	0.086	0.606^**^	0.204	0.264	−0.034	1.000	
	Interferon‐γ	0.180	0.741^**^	0.423	0.810^**^	0.755^**^	−0.207	0.403	0.252	−0.073	−0.364	0.300	1.000

Data are presented as Spearman's rho correlation coefficient. Significant correlations are presented with *p < 0.005; **p < 0.001.

CSC = central serous chorioretinopathy, GM‐CSF = granulocyte‐macrophage colony‐stimulating factor, IL = interleukin, TNF‐α = tumour necrosis factor‐α, VEGF = vascular endothelial growth factor.

## Discussion

The role of cytokines in CSC and their potential mechanism of action are still not well established. Cytokines were suggested to be involved in ocular diseases that contribute to choroidal abnormalities (Schellevis et al. 2018; Weinstein & Pepple 2018). To our knowledge, our study is the first comprehensive analysis of various cytokines in the plasma of patients with acute and chronic CSC as compared with healthy individuals.

We noted differences in plasma levels of IL‐6, IL‐10, IL‐12 p70 and VEGF between the three study groups. Some of the analysed cytokines are involved in retinal and choroidal hyperpermeability, while the role of hyperpermeability of the thickened choroid in the pathogenesis of CSC has been emphasized in several recent studies (Kim et al. 2011; Chung et al. 2016; Sakurada et al. 2018). Interleukin‐6 (IL‐6) and VEGF alter the junction integrity and downregulate occludin and zonula occludens‐1 (Behzadian et al. 2003; Murakami et al. 2012; Liu et al. 2016; Yun et al. 2017). Moreover, IL‐6 activates VEGF production by the STAT3 protein (Yun et al. 2017), which causes increased vascular permeability and angiogenesis (Mesquida et al. 2014). Interleukin‐12 (IL‐12) regulates angiogenesis (Zhou et al. 2016) and induces T‐helper type 1 (Th1)‐specific immune responses (Manetti et al. 1993).

Interleukin‐10 (IL‐10) is an essential anti‐inflammatory cytokine responsible for negative regulation of immune responses to microbial antigens (Rutz & Ouyang 2016). It is known to exert multidirectional and complex actions, including the immune regulation of autoimmune diseases (Jung et al. 2019). It is generally classified as an anti‐inflammatory cytokine; however, it is important to note that its pro‐inflammatory activity in vivo has also been reported (Sharif et al. 2004; Mühl 2013).

Some investigators showed that serum IL‐1β, IL‐6, IL‐8, IL‐10, IL‐17, IL‐22, IL‐23 and TNF‐α play an important role in uveitis, and their function is related to pathologic Th17 cells (Zelazowska‐Rutkowska et al. 2017; Weinstein & Pepple 2018). Experimental studies have reported Th17 cells to be the key mediators of inflammatory eye disease (Weinstein & Pepple 2018). A relationship between the systemic upregulation of cytokines in patients with neovascular AMD has also been postulated (Zehetner et al. 2014; Faber et al. 2015; Nassar et al. 2015), but the results of recent studies indicate that AMD is related to dysregulation of immune intraocular factors, whereas plasma cytokine levels are not elevated, as compared with controls (Fauser et al. 2015; Agrawal et al. 2019). Moreover, the role of inflammation and the involvement of intraocular cytokines in macular oedema have been postulated (Daruich et al. 2018).

We identified only one report in the literature describing the involvement of plasma cytokines (VEGF and IL‐8) in the pathophysiology of CSC (Lim et al. 2010). Lim et al. revealed no differences in plasma IL‐8 and VEGF levels between CSC and controls (Lim et al. 2010), but our study showed a significant upregulation of plasma IL‐6, IL‐10 and IL‐12 p70 levels in patients with chronic CSC. Contrary to Lim et al. (2010), our results support the association between plasma cytokine dysregulation and CSC.

More data are available on AH than on plasma cytokine levels in CSC, but the results are inconsistent. Terao et al. (2018) noted that AH IL‐6 and IL‐8 levels were upregulated in patients with chronic CSC. Some investigators did not find any difference in IL‐6, IL‐8, monocyte chemo‐attractant protein‐1 and VEGF levels in AH between patients with CSC and controls (Lim et al. 2010; Shin & Lim 2011). However, it may be due to the fact that AH in control groups in those studies was obtained mostly from young patients with cataract, which can indicate the coexistence of some other general or eye diseases (Lim et al. 2010; Shin & Lim 2011). In line with the study by Terao et al. (2018), we hypothesize that intraocular and systemic upregulation of IL‐6 levels may be involved in the pathophysiology of CSC.

Surprisingly, we noted a lower plasma VEGF concentration in patients with chronic CSC, as compared with healthy individuals. The cytokine was downregulated only in chronic CSC, which is consistent with the findings reported by Shin & Lim (2011). However, unlike in our study, they analysed the cytokine profile in AH samples. No differences in plasma VEGF levels between patients with CSC and controls were reported by Lim et al., but the sample size was small (Lim et al. 2010). The VEGF family includes VEGF‐A, VEGF‐B, VEGF‐C, VEGF‐D, VEGF‐E and placental growth factor (PlGF). Among all factors, the overexpression of PlGF and VEGF‐A levels increases vascular permeability (Witmer et al. 2003). In our opinion, altered ocular levels of pro‐inflammatory cytokines and growth factors may have significant implications for increased vascular permeability and development of CNV in the course of CSC. However, in our study, patients with chronic CSC, which is associated with a higher incidence of CNV, had lower VEGF plasma levels than controls. This may indicate an imbalance between the levels of VEGF and other growth factors, but such a hypothesis requires further research. Nonetheless, our results are in line with the study by Spaide (2015) and Sacconi et al. (2019). They hypothesized that CNV in CSC is due to proliferation of new vessels during arteriogenesis, which is characterized by dilation of the existing vascular channels and is independent of VEGF (unlike angiogenesis, which is highly VEGF dependent) (Schierling et al. 2009; Wu et al. 2010).

Based on OCT angiography, Shiragami et al. (2018) reported CNV in 15.6% of cases with acute CSC and in 21.8% of those with chronic CSC. Bousquet et al. (2018) revealed the prevalence of CNV in 35.6% of patients with chronic CSC, with flat irregular pigment epithelial detachment. Sacconi et al. (2019) considered arteriogenesis to be the central driving force of CNV development in the course of CSC, because despite noting some clinical response after anti‐VEGF therapy, the density of CNV vessels did not change on OCT angiography. Downregulated plasma VEGF levels in CSC observed in our study may explain the unsatisfactory effect of intravitreal anti‐VEGF treatment in patients with CSC, which was reported in meta‐analyses (Chung et al. 2013; Ji et al. 2017). In neovascular AMD, the same patients may also show a weak or no reaction to treatment due to nonresponsiveness or tachyphylaxis (Żuber‐Łskawiec et al. 2019).

Interestingly, in our study, IL‐6 was the only cytokine upregulated in acute and chronic CSC. Multifunctional IL‐6 is involved both in the normal acute inflammatory response and in the detrimental low‐grade systemic chronic inflammatory process (Morieri et al. 2017). Apart from pleiotropic activity and the key role in host defence against environmental stress, IL‐6 also increases vascular permeability (Mesquida et al. 2014). Rochfort et al. noted that this cytokine decreases the expression of claudin 5, occludin and vascular endothelial cadherin in human brain microvascular endothelial cells. They also demonstrated that IL‐6 downregulates the expression of tight junction proteins and interendothelial adherence, thus increasing paracellular permeability (Rochfort et al. 2014). As stated above, hyperpermeability of the choriocapillaris and thickened choroid plays an essential role in the pathogenesis of CSC (Kim et al. 2011; Sakurada et al. 2018). In our study, a positive correlation was noted between IL‐5, IL‐6, IL‐12 and mean CT only in acute CSC. The mechanism of vasodilation of large choroidal vessels in CSC has not been fully elucidated. Given the association between cardiovascular disease and risk factors for vascular eye diseases, we speculate that increased levels of pro‐inflammatory cytokines lead to abnormal endothelium‐dependent vasodilation (Sitia et al. 2010; Karska‐Basta et al. 2011).

It has been shown that glucocorticoids are involved in the pathogenesis of CSC, and glucocorticoid receptors are expressed both in the choroid and retina (Zhao et al. 2010; Brinks et al. 2018). In rats and humans, corticosterone may cause choroidal thickening, a feature typical for patients with CSC (Zhao et al. 2012). When psychologically stressed (a known risk factor for CSC), the body produces stress hormones such as cortisol, which are able to trigger IL‐6 release into the circulation (Clark et al. 2019). Moreover, glucocorticoids were shown to enhance IL‐6‐dependent expression of pro‐inflammatory genes by inhibiting the suppressor of cytokine signalling 3, a physiological mechanism that controls acute inflammatory response (Dittrich et al. 2011). Inflammation is recognized as one of the key factors in the pathogenesis of AMD, with glucocorticoid treatment showing no major benefit (Geltzer et al. 2013). We speculate that a similar mechanism may play a role in CSC (and particularly in chronic CSC, as shown in our study). The disease is associated with increased levels of some pro‐inflammatory cytokines, but the use of steroid treatment paradoxically worsens rather than alleviates symptoms. It is not clear whether steroids and cytokines exert their effects independently of or in combination with one another in patients with CSC.

Higher levels of IL‐6 and IL‐12 with pro‐atherogenic properties were seen in patients with CSC and a thicker choroid (Sprague & Khalil 2009). It is difficult to explain the positive correlation between CT and plasma IL‐5 levels in patients with acute CSC, because IL‐5 suppresses VEGF‐induced angiogenesis through STAT5 signalling (Bucher et al. 2018). Usually, a decrease in VEGF in intraocular fluids due to intravitreal anti‐VEGF therapy leads to a reduction in CT, for example in patients with diabetic retinopathy (Kniggendorf et al. 2016). However, this phenomenon requires further investigation.

A persistent increase of plasma cytokine levels has been reported not only in patients with autoimmune diseases but also in those with a history of cardiovascular disease (Morieri et al. 2017) and vascular dysfunction (Sprague & Khalil 2009), such as atherosclerosis, abdominal aortic aneurysm, varicose veins and systemic hypertension (Wenzel et al. 2016).

The results of our study confirmed positive correlations between systemic hypertension and plasma IL‐8, IL‐6 and TNF‐α levels in chronic CSC. Growing evidence indicates that the immune response affects the pathogenesis of hypertension (Wenzel et al. 2016). Also, T cells contribute to the development of this condition. The expression of specific transcription factors and the profile of cytokines produced by CD4^+^ T cells are the basis for classification into 4 major subsets: Th1, Th2, Th17 and regulatory T cells (Wenzel et al. 2016). On the other hand, in our study, controls with hypertension did not have higher cytokine levels than individuals without hypertension. However, any general conclusions are limited by the small size of the analysed group. Nonetheless, based on available literature regarding patients with hypertension without CSC, we may speculate that cytokines that were shown to be elevated in our study are also elevated in the general population of patients with hypertension. The cytokines IL‐6 (Zhang et al. 2012), IL‐8 (Marek‐Trzonkowska et al. 2015) and TNF‐α (Puszkarska et al. 2019) are considered to play an important role in the pathogenesis of hypertension, but their excessive levels were also reported to negatively affect the function of numerous organs. Chae et al. (2001) reported increased blood pressure as a stimulus for inflammatory response and hypothesized this to be a mechanism underlying the role of hypertension as a risk factor for atherosclerotic disease. Our study, which revealed elevated levels of selected cytokines in patients with hypertension and chronic CSC, suggests that hypertension may impact choroidal abnormalities in the course of this long‐lasting eye disease.

The most important limitations of our pilot study include the fact that the samples were obtained only from plasma and that the study was performed at a single time‐point.

In conclusion, we speculate that altered plasma cytokine levels, including downregulation of VEGF and upregulation of IL‐6, IL‐10 and IL‐12 in patients with chronic CSC may reflect an indirect role of these factors in vascular changes observed in the course of this disease. Our results suggest a previously unknown role of plasma cytokines in the pathogenesis of CSC. However, it remains unclear whether abnormalities in intraocular or systemic cytokine levels are more involved in disease development. There is also a possible association between the inflammatory response in CSC and systemic vascular changes. Therefore, further research is needed to confirm this hypothesis.
